# Macrophages: friend or foe in diabetes pathogenesis and therapy

**DOI:** 10.3389/fimmu.2025.1625391

**Published:** 2025-12-15

**Authors:** Rachel N. Grimes, Marco Orecchioni, Estefania Quesada-Masachs

**Affiliations:** 1 Medical College of Georgia, Augusta University, Augusta, GA, United States; 2 Immunology Center of Georgia, Augusta University, Augusta, GA, United States; 3 Department of Pharmacology and Toxicology, Augusta University, Augusta, GA, United States; 4 Diabetes Research Institute, University of Miami Miller School of Medicine, Miami, FL, United States

**Keywords:** type 1 diabetes, type 2 diabetes, macrophages, beta cells, inflammation, adipose tissue, tissue resident macrophages

## Abstract

Macrophages play a key role in the pathogenesis of both type 1 (T1) and type 2 (T2) diabetes, influencing disease initiation and progression through distinct mechanisms reflective of their divergent etiologies. In type 1 diabetes, an autoimmune condition characterized by the destruction of insulin-producing pancreatic beta cells, macrophages are part of the inflammatory response, which initiates insulitis and leads to pancreatic beta cell death. Conversely, in type 2 diabetes, which is primarily driven by insulin resistance and metabolic dysregulation, macrophages infiltrate the adipose tissue and contribute to a chronic state of low-grade inflammation. They therefore have a dual effect, driving diabetes by facilitating autoimmunity and perpetuating metabolic dysfunction and meta-inflammation. Macrophages infiltrate the pancreas in both patients with T1 and T2 diabetes. However, we cannot assume that an increase in the number of macrophages in the pancreatic infiltrate is a pathological feature of diabetes. Macrophages are also known to participate in embryonic islet development and to contribute to pancreatic regeneration and islet remodeling. It is possible that their function at the site of inflammation is part of the recovery process rather than the attack itself. Macrophages express high plasticity, which results in high functional heterogeneity both in steady-state and in pathological conditions, with a continuum of extreme phenotypic and functional states. Activated macrophages release inflammatory mediators, which amplify the autoimmune response and foster an environment that may contribute to beta cell destruction in type 1 diabetes. Recent studies have shown that lipid accumulation and metabolic dysfunction can contribute to macrophage activation, a theory that links obesity to enhanced inflammatory responses and insulin resistance, which is central to the pathophysiology of T2 diabetes. Targeting macrophage polarization and function presents a promising therapeutic strategy for mitigating disease progression in both types of diabetes. Understanding the intricate roles of macrophages in T1 and T2 diabetes is crucial for developing effective interventions to modulate the immune response and improve overall metabolic health. Here, we review the current knowledge of the heterogeneity and origin of macrophages, their role at the sites of inflammation in T1 and T2 diabetes, and their potential for therapeutic strategies.

## 1 Introduction

Diabetes is a major public health concern, affecting 38.4 million Americans, 11.6% of the U.S. population as of 2021 (1). Type 1 diabetes (T1D) and type 2 diabetes (T2D) are distinct diseases that converge in their disruption of glucose metabolism. T1D and T2D prevalence and incidence have been progressively and significantly increasing in youth for reasons still unclear (2, 3). T1D is an autoimmune disease caused by the selective immune mediated destruction of the insulin-producing beta cells, leading to a severe deficiency of insulin (4). Currently, there are an estimated 9.5 million people living with T1D worldwide (2 million only in the U.S.), a 13% increase from 2021 (5). T2D is a metabolic disease characterized by insulin resistance, hyperinsulinemia, and hyperglycemia and its development is strongly associated with obesity among other factors (6). T2D is much more common than T1D, and it is projected to affect 1 in 8 adults, approximately 853 million people, by 2050 (7). Insulin-sensitive tissues become less responsive to insulin, and, to facilitate glucose uptake in peripheral tissues, beta cells in the pancreas compensate by increasing insulin production, a process known as "compensatory hyperinsulinemia". However, there is a chronic deficiency in glucose uptake and impaired insulin action, particularly in the liver, skeletal muscle, and adipose tissue. This situation causes persistent hyperglycemia, which leads to endothelial damage through increased oxidative stress, ultimately enabling many of the complications associated with diabetes, such as nephropathy, retinopathy, and neuropathy (8).

Increasing evidence implicates macrophages as key mediators in the pathogenesis of both T1D and T2D, largely through their infiltration into adipose tissue and pancreatic islets (9). Macrophages are highly plastic innate immune cells essential for tissue homeostasis, host defense, and inflammation regulation. They play diverse roles in both innate and adaptive immunity, including phagocytosis, antigen presentation, tissue remodeling, and immune resolution (10). Their function is context-dependent, allowing them to contribute to both disease progression and resolution (11). In adipose tissue, macrophage infiltration is observed in people with obesity, particularly in individuals with T2D. Adipose tissue macrophages contribute to the development of insulin resistance by promoting chronic low-grade inflammation or “meta-inflammation” (12). Their presence in adipose tissue correlates with insulin resistance and is considered a key contributor to the development of T2D (9). A growing body of evidence sheds some light on the mechanisms that link T1D and inflammation of the adipose tissue, for instance, in the context of obesity (12).

Macrophages are present in the pancreas of individuals without diabetes. They are required for normal beta cell development during embryogenesis and, later in life, support beta cell replication and local tissue repair (13, 14). However, increased infiltration has been observed in pancreatic islets from patients with both T1D and T2D (15–17). Macrophages, both monocyte-derived and tissue-resident, play intricate roles in the pathogenesis of T1D and T2D. This review explores the complex and sometimes paradoxical roles of macrophages in diabetes, outlines macrophage origins and polarization, their involvement in pancreatic islets and adipose tissue during diabetes pathogenesis, and summarizes current and emerging therapeutic strategies that target macrophage function to prevent or mitigate disease initiation or progression.

## 2 Macrophage heterogeneity: origin

Macrophages in tissues exhibit impressive heterogeneity in their populations and functions across different tissues, contributing to both homeostasis and disease (18). Recent evidence indicates that most adult tissue macrophages originate during embryonic development. However, historically, macrophages were considered as fully differentiated cells replenished by circulating monocytes and stimulated by macrophage colony-stimulating factor (M-CSF) as a part of the mononuclear phagocyte system (19). It has been demonstrated that distinct macrophage populations can coexist in a similar microenvironment, raising the possibility that differences in origin and ontology play important roles in determining later-stage differences (20, 21). Currently, it remains unclear whether macrophages of distinct origins are functionally equivalent or possess distinct roles under steady-state conditions (18).

Monocytes are released from bone marrow into the circulation and migrate to tissues around the body or to the spleen, where they are stored as immature precursors (22). Extravasation of monocytes through the endothelium coincides with differentiation into mature phagocytes, such as macrophages or dendritic cells. Tissue-resident macrophages arise prenatally from embryonic progenitors in the yolk sac and fetal liver (20, 23–26). These tissue-resident macrophages self-renew within the organ and are locally maintained, with minimal contributions from circulating monocytes (20, 26). Tissue-specific macrophages are found in various tissues, ranging from osteoclasts in the bone to alveolar macrophages in the lungs. In these niches, they exhibit distinct gene profiles and perform tissue-specific functions that complement their roles in phagocytosis, immune surveillance, and antigen presentation (27). Tissue conditions program differentiation of the resident macrophages (28).

The origin of human adipose tissue macrophages remains unclear. Studies in mice suggest that they predominantly originate from circulating monocytes rather than through the proliferation of resident macrophages (29). Adipocytes produce chemoattractants specific for monocytes and macrophages (30) and can also produce M-CSF (31). However, some evidence suggests the presence of tissue-resident macrophages (32). Similarly, pancreas-resident macrophages are believed to arise from both embryonic yolk sac precursors and circulating monocytes, with their origin influenced by local developmental cues (23, 33).

## 3 Macrophage heterogeneity: polarization

Macrophage functions and mechanisms are diverse, ranging from pro-inflammatory actions that may promote tissue damage to anti-inflammatory effects that can promote tissue repair and modulate the immune response. Their effects can vary between local impacts, for example in the pancreatic islets, or systemic effects, for example in adipose tissue (34).

Macrophages have been classically characterized as having either a proinflammatory M1 or immunoregulatory M2 phenotype. The M1/M2 paradigm provided a useful framework to study immune responses; however, this is reductive to their plasticity (35, 36). During the years, various terms and definitions have emerged in the literature to describe macrophage activation and polarization more comprehensively, such as regulatory macrophages (Mreg), highlighting their immunomodulatory roles (37); Mox macrophages, which are specifically induced by oxidized lipids and associated with oxidative stress responses (38); M4 macrophages, differentiated in the presence of platelet factor 4 (CXCL4/PF4), and defined by a unique transcriptomic profile (39). Mantovani et al. further subdivided the M2 macrophages into subcategories (M2a, M2b, and M2c) depending on their stimuli and functional characteristics (40). Several reviews examined and discussed these aspects of macrophage polarization thoroughly (41–43). However, this expanded classification can still be considered an oversimplification, based often on *in vitro* observations, and may not properly represent the macrophage phenotypes observed *in vivo* (36).

**Figure 1:**
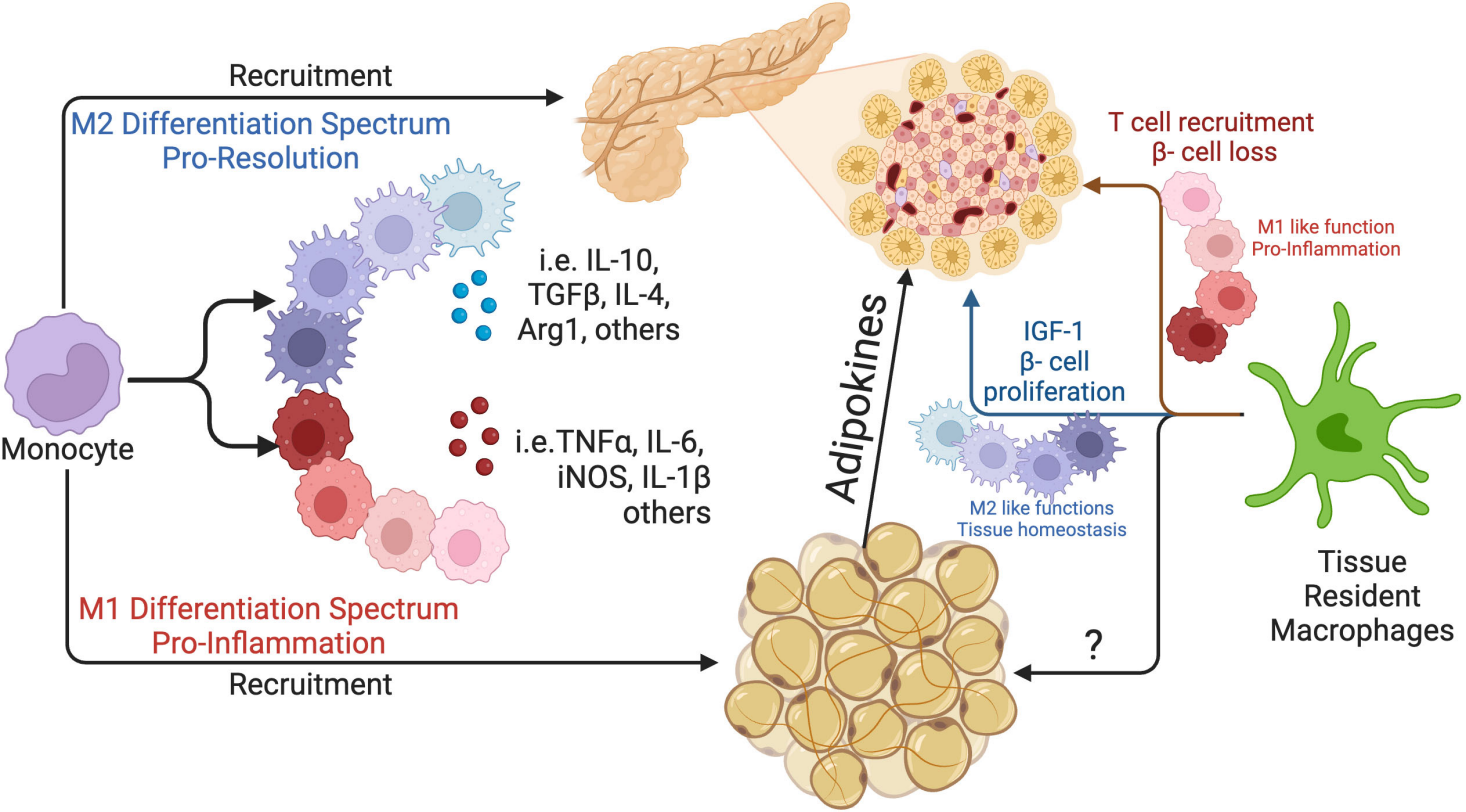
Macophages in the pancreas and in the adipose tissue. Illustration of both origins of macrophages present in the pancreas and in the adipose tissue: peripheral vs tissue resident macrophages generated during embryonic life. The figure is also illustrating how adipokines secreted by the adipose tissue can impact the pancreatic environment. Additionally, macrophages exhibit high functional plasticity and respond to their local microenvironment. In both pancreas and in the adipose tissue, macrophages will further differentiate acquiring a more regulatory or a more inflammatory profile, based on local stimulus.

The tissue microenvironment is complex with the simultaneous presence of several stimuli, and the high plasticity of macrophages allows many intermediate functional states (**Figure 1**). They can contribute to both protective and pathological processes in tissues. Moreover, evidence indicates that polarization states can be dynamically switched in response to environmental stimuli (44), further challenging the rigid notion of two distinct phenotypes. Contemporary consensus suggests that macrophage polarization exists along a continuum from pro-inflammatory to anti-inflammatory states, driven by cytokine secretion, transcriptional mechanisms, and metabolic phenotypes, enabling these cells to exhibit plasticity in response to environmental cues (40, 41). Therefore, macrophage polarization is marked by distinct functional phenotypes, adding to their adaptability in diverse physiological scenarios (45, 46). At one end of the polarization spectrum, pro-inflammatory M1 macrophages are linked with antimicrobial defense and inflammatory responses, while alternatively activated M2 macrophages are involved in tissue repair, immunoregulation, and trophic functions (47).

In adipose tissue, macrophages mostly appear during embryonic development and polarize into distinct phenotypes in response to environmental stimuli, such as adiposity (18). Among these resident cells, a subset characterized by CD206 expression plays a role in regulating systemic glucose homeostasis (48). These macrophage subsets have been shown to modulate the adipose tissue dynamics by limiting the proliferation of adipocyte progenitors through the downregulation of TGF-ß signaling. Ablation of these CD206+ macrophages in mice increases the number of smaller adipocytes and improves systemic insulin sensitivity (48). For the adipose tissue macrophages, polarization is not rigidly categorized into M1 or M2 phenotypes as previously believed. It is more of a dynamic process that can be reversed under physiological and pathological conditions (49). That may explain why polarization patterns and their metabolic effects differ across studies, with conflicting data on whether pro-inflammatory ATMs directly impair systemic insulin sensitivity or reflect compensatory remodeling (33, 50, 51). Single-cell analyses have revealed that adipose tissue macrophages display mixed characteristics and functions between M1 and M2, showing high heterogeneity based on obesity status (29, 52–54). Intriguingly, specific subsets of TREM2-expressing cells arise and accumulate in the adipose tissues. These macrophages are also named Lipid Associated Macrophages (LAM) and are identified in mice and humans across multiple organs that undergo loss of metabolic homeostasis (29). This includes subsets like CD9+ ATMs, and perivascular macrophages, all of which are enriched in obesity and contribute to adipose tissue inflammation and insulin resistance (29, 53). In the liver of patients with metabolic dysfunction-associated steatohepatitis (MASH), TGF-β, among other molecules, has been identified as a crucial regulator of disease-associated expansion of TREM2 + macrophages (55). The polarization of ATMs is regulated by various signals leading to the activation of pathways such as toll-like receptor (TLR)-4, MAPK, and NF-κB signaling, promoting a pro-inflammatory phenotype. In contrast, molecules such as adiponectin, peroxisome proliferator-activated receptor-γ (PPAR-γ), and IL-4 promote an anti-inflammatory phenotype in ATMs (56, 57). Their balance likely controls the inflammatory status and homeostasis of the adipose tissue.

Environmental signals, including cytokines, chemokines, and microbial products, finely orchestrate macrophage polarization, leading to specialization tailored to specific tissue microenvironments (21, 34). The dynamic function and polarization of macrophages make them pivotal players in maintaining tissue homeostasis, regulating immune responses, and driving physiological processes across organ systems. It has become evident that macrophage phenotype and function are dynamic and quickly respond to changes in the tissue and in the context of disease-specific factors (35). Fast changes in their phenotype could be the result of de-differentiation or of the migration of a new population of macrophages into the tissue (42). Phenotype shifts in the macrophage population over time are often associated with pathological processes. A more comprehensive characterization and understanding of macrophage phenotypes in adipose tissue and the pancreas is still warranted. Overall, macrophages are essential cells in tissue homeostasis and in the context of inflammation, whether this be recruited macrophages that may act to promote inflammation in response to local challenges or resident macrophages that may be important in the promotion of repair responses (58).

## 4 Macrophages in the pancreas: their role in the development of islets

During embryonic development, macrophages derived from the yolk sac accumulate in the pancreas, particularly near islets, which contain insulin-producing beta cells (50). Pancreatic resident macrophages play an important role in the early development of islets, controlling the morphogenesis and initial function of beta cells (20, 33). Studies in mice have demonstrated that the induced deficiency of macrophages can impair pancreatic islet development and the expansion of beta cells (13, 20). Mice with a spontaneous mutation in the colony-stimulating factor 1 (CSF-1) protein lack macrophages from birth. They have a marked reduction in islet mass and respond poorly to glucose challenge (28). However, research conducted in adult mice has shown that the absence of macrophages later in life does not significantly impact islet function, suggesting that their relevance lies primarily in islet development rather than in the maintenance of islet function (59). Additionally, they contribute to beta cell homeostasis, secreting growth factors such as insulin-like growth factor 1 (IGF-1), which is produced in response to beta cell death, stimulating beta cell proliferation and viability (60).

Pancreatic resident macrophages exhibit diverse phenotypes depending on their anatomical location (**Table 1**). In adult mice, islet resident macrophages are characterized by a high proinflammatory profile, with elevated expression of MHC class II and the secretion of various cytokines and chemokines, including TNF-α and IL-1β (61). These macrophages can sense blood products, capture granules transferred from adjacent beta cells and present peptides to autoreactive CD4 T cells, and are ready to initiate an immune response within the pancreas even in steady-state conditions (61, 62). Exocrine pancreatic macrophages, in contrast, typically exhibit an immunoregulatory M2-like phenotype and have lower phagocytic capacity than their islet counterparts (33, 63). Recent research by Ying et al. has identified two distinct macrophage subsets within pancreatic islets under steady-state conditions. The F4/80low CD11c+ population was found to be enriched within the islets, while the F4/80hi CD11c- macrophages were primarily located in the peripheral islet area (9, 64).

**Table 1:** Defined distinct subsets of adipose and pancreatic macrophages in homeostasis and disease.

Tissue	Context	Subset	Key markers	Location / origin	Function	Species	Ref.
White Adipose Tissue (WAT / scWAT / eWAT)	Homeostasis	Parenchymal ATMs (pATMs)	LYVE1-low, MHCII-low, CX3CR1, CCR2, Fcrg4 (CD16.2)	Parenchyma; monocyte-derived	Antigen presentation; immune surveillance	Mouse	(109)
	Homeostasis	Capsular ATMs (cATMs)	LYVE1-high, MHCII-low, CD209b+, TIM4+	Capsular region; embryonic	Tissue remodeling; barrier maintenance	Mouse	(109)
	Homeostasis / Obesity	Septal ATMs (sATMs)	CD209b+, LYVE1+, CD206+, TIM4+, TGFβ1	Septa near CD26^+^ ASCs; embryonic	Promote white adipogenesis; restrain beige adipocyte formation via TGFβ1	Mouse + Human	(109)
	Obesity	LAMs (Lipid-associated macrophages)	TREM2, CD9, APOE, CD11c	Crown-like structures; monocyte-derived	Lipid clearance; metabolic inflammation; insulin resistance	Mouse + Human	(29, 53)
	Obesity	Proliferating ATMs (crown-like macrophages)	Ki67^+^, CD11c^+^, CD68^+^	Surround dead adipocytes (crown-like structures)	Phagocytose debris; secrete TNFα, IL-1β; sustain inflammation	Mouse + Human	(53, 106)
Pancreas	Homeostasis (embryonic)	Embryonic / yolk-sac–derived macrophages	F4/80+, CD11b+	Peri-islet; embryonic yolk sac origin	Regulate islet morphogenesis; support β-cell expansion	Mouse	(20, 28, 33, 50)
	Homeostasis (adult)	Islet resident macrophages	F4/80+, CD11c+,MHCII+ CX3CR1+ CD206-	Intra-islet; embryonic + monocyte	Antigen presentation; β-cell surveillance	Mouse + Human	(33, 61)
	Homeostasis	Exocrine macrophages	F4/80-high, CD11c–, CD206+, IL-10	Exocrine tissue; embryonic	Immunoregulatory; tissue integrity	Mouse	(33, 63)
	Homeostasis	Mac-1	Cst3, Cd74, Prdx1, Apoe	Resident	Homeostatic antigen-presenting macrophage	Mouse	(90, 91)
	Activation (autoimmunity)	Mac-2 (pro-inflammatory)	Tnf, Ccl3, Atf3	Islets	Cytokine production; T-cell activation; initiate inflammation	Mouse	(90, 91)
	Intermediate	Mac-3	H2-Ab1, Cd74, Ifitm3, Cxcl9	Islets	Antigen-presentation transitional subset	Mouse	(90, 91)
	Beta-cell apoptosis / tolerance	Mac-4 (e-Macs)	Cd9, Lgals3 (Gal-3), Mertk, Igf1	Islets; efferocytosis-induced	Secrete IGF-1; induce CD4^+^ T-cell anergy via IGF-1/IGF1R; protect against autoimmunity	Mouse + Human	(90, 91)
	Remodeling	Mac-5 (proliferating)	Mki67, Birc5,Stmn1	Islets; cycling	Maintain macrophage pool; regeneration	Mouse	(90, 91)
	Diabetes	Activated / infiltrating macrophages	CD68,HLA-II, MHCII-high, CD11c+, TNFα, IL-1β, Nos2	Intra-islet and peri-islet	Promote β-cell apoptosis; initiate insulitis; activate T cells, correlate with disease	Mouse + Human	(59, 66, 69, 81, 82)
	Beta-cell repair	IGF-1 secreting macrophages (similar to eMacs)	Igf1, Mertk, Cd206	Islet macrophages responding to β-cell death	Stimulate β-cell proliferation and viability; maintain homeostasis	Mouse + Human	(60, 91)
	Diabetes (Human)	CD206^+^ vs CD206^-^ macrophages	CD206 (MRC1)	Islet and exocrine regions	CD206^+^: anti-inflammatory; CD206^-^: pro-inflammatory; potential ontogenic difference	Human	(92)

ASCs, adipocyte stem cells; ATM, adipose tissue macrophages; eWAT, Epididymal white adipose tissue; MHCII, Major Histocompatibility Complex class II; HLA-II, Human Leukocyte Antigen class II; IGF-1, insulin-like growth factor 1; scWAT, subcutaneous white adipose tissue.

Pancreatic resident macrophages are constantly uptaking microparticles, especially those in close proximity to the islets, and they are reactive to the local microenvironment (63, 65). The intrinsic heterogeneity of macrophages may explain their dual role as key players in homeostasis and inflammation within the pancreas.

## 5 Macrophages in the pancreas: their role in the development of diabetes

Macrophages are the predominant immune cell population in the pancreas, even in healthy individuals (15, 66). Macrophages in islets contribute to pancreatic regeneration and islet remodeling, and are relevant in maintaining islet homeostasis (14). However, macrophages and other immune cells have been reported to infiltrate the pancreas in patients with T1D and T2D, suggesting their involvement in disease pathogenesis (15, 17, 66–68). Macrophages have a dual role in T1D and T2D, participating in both beta cell death and beta cell regeneration (69).

In patients with T1D, there is an important loss of beta cell mass caused by the islet immune attack. Several types of immune cells infiltrate the islets, a key phenomenon called insulitis that is much more modest in humans than in animal models of autoimmune diabetes, such as NOD mice (70, 71). Islet infiltration by immune cells in patients with T1D is highly heterogeneous between donors and within the islets of the same donor, and its distribution may be more lobular (71–73). In preclinical models, most often NOD mice, numerous interventions that were effective in these models failed to demonstrate the same efficacy in human T1D trials due to data interpretation and the complexity and heterogeneity of the disease in humans (74). CD8 T cells are the principal T cell type infiltrating the islets in T1D patients (15, 71, 75, 76), and antigen-specific T cells (CTLs) have been shown to kill human beta cells *in vitro* (77). Although CTLs are present in healthy individuals, they are believed to be key players in T1D pathogenesis (78). Macrophages can initiate inflammation, possibly by controlling the initial entrance of T cells into the islets and the progressive loss of insulin-secreting beta cells (59). A large body of existing evidence from studies conducted in the non-obese diabetic (NOD) mouse model suggests that macrophages play a major role in the initiation of the autoimmune process by being instrumental in exposing the islets to the circulating diabetogenic T cells (28). Additionally, macrophages contribute to the autoimmune response against pancreatic beta cells by releasing pro-inflammatory cytokines and promoting cytotoxic T cell activation (79).

**Figure 2:**
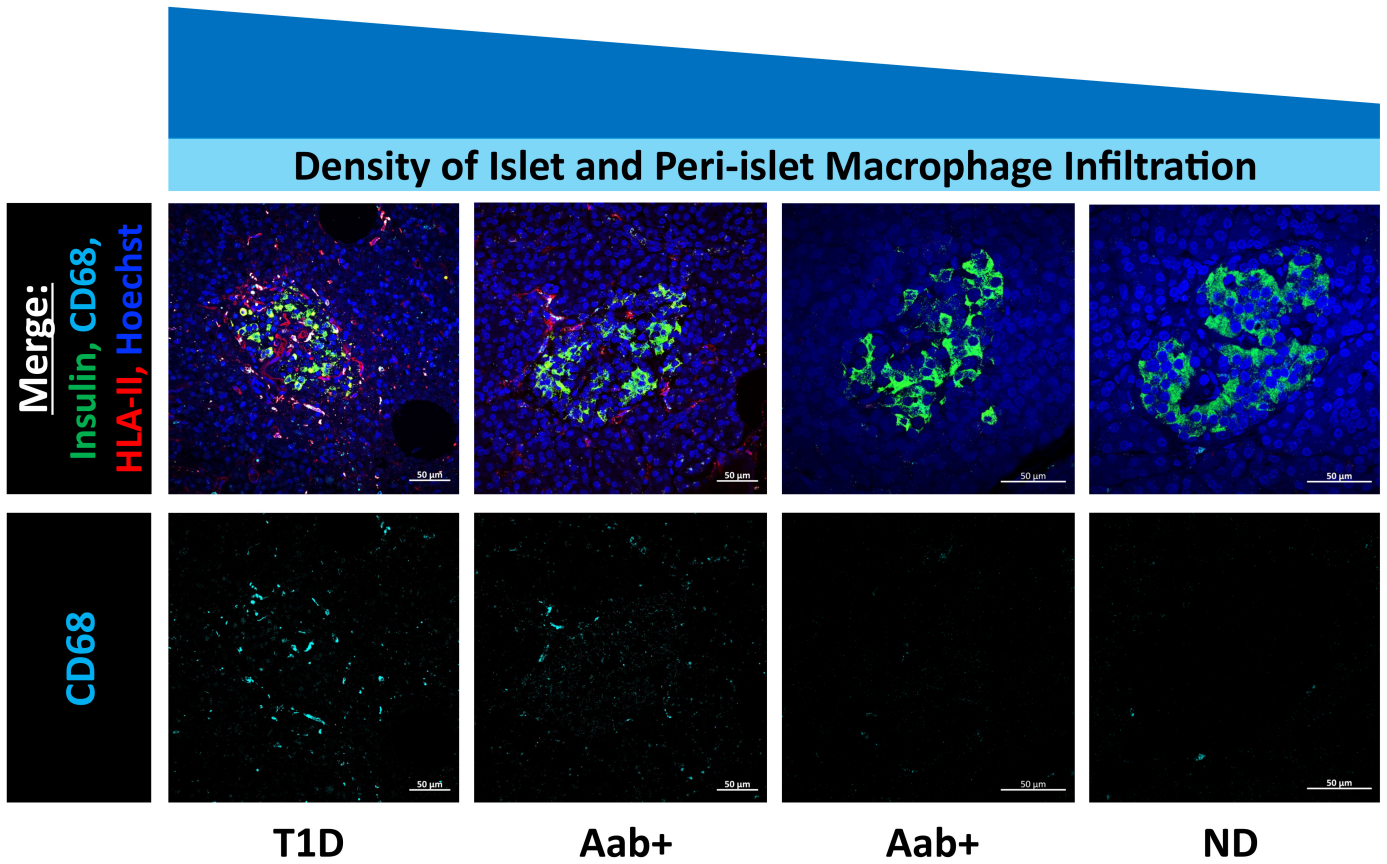
Pancreatic islet and peri-islet infiltration of macrophages. Representative high-resolution confocal images acquired with the Zeiss laser scanning confocal microscope LSM880 with an oil 40x (1.4 NA) objective (Dr. Quesada-Masachs lab). The upper line of images shows a merged combination of staining with Hoechst in dark blue, insulin in green, CD68 in turquoise and HLA class II in red (scalebars, 50µm). The row below shows single staining with CD68 in turquoise to appreciate the detail on the abundance of the macrophage infiltration. Each image shows a single islet used as a representative image of donors with different levels of islet infiltration by macrophages and different disease states, being the highest infiltration observed in the patient with type 1 diabetes (T1D) followed by one of the patients with autoantibodies (Aab+), and the lowest infiltration in the other Aab+ donor and the non-diabetic (ND). Notice that HLA class II expression (in red) was also the highest in those T1D and Aab+ cases, probably indicating a more activated state of those macrophages actively infiltrating the islet.

Macrophages are consistently identified as one of the largest populations of immune cells infiltrating the islets in T1D, and their numbers are more abundant than those observed in individuals without diabetes (15, 66, 80, 81). The triggers leading to the pro-inflammatory microenvironment in the pancreas prior to the development of T1D are still not fully elucidated; however, macrophage activation may play a role. Islet resident macrophages can sense both local and blood-derived molecules and, even in normal conditions, can induce an immune response (61). In mouse models of autoimmune diabetes, macrophages infiltrate the islet and peri-islet regions before the onset of disease (82). Furthermore, early depletion of islet resident macrophages in NOD mice results in diminished lymphocyte infiltration and a lower incidence of autoimmune diabetes (59). To this extent, it has recently been reported that there is an increased macrophage infiltration in the pancreas of some autoantibody-positive patients (considered at risk of developing T1D), suggesting that this may be a relevant early phenomenon in T1D chronology; hence, macrophages are important for disease initiation (81) (**Figure 2**).

Similarly, in T2D donors, macrophage infiltration of pancreatic islets is increased (16, 17, 83). Islet macrophages in T2D have a polarity shifted towards M1 (84–86) and contribute to the impairment of beta cell function and insulin secretion (9, 85). Interestingly, some patients with T2D even fulfill the definition of insulitis proposed for patients with T1D (67, 83). Beta cell death and dysfunction in T2D is influenced by chronic elevation of glucose and free fatty acid levels, and by their crosstalk with inflammatory M1-like macrophages (69). The T2D exocrine pancreas can present signs of chronic inflammation (fibrosis), but macrophage density and polarization don’t seem to differ from controls (87). In obese individuals, macrophages in islets are not infiltrating at higher numbers, but exhibited a higher inflammatory profile, reinforcing the concept that obesity is a potential initiating factor in the pro-inflammatory cascade that triggers T2D (88).

Poor metabolic control in T1D and T2D can cause high blood glucose levels and circulating saturated fatty acids, which can induce pancreatic beta cell dysfunction, beta cell apoptosis, and lead to the release of ATP, chemokines, and apoptotic cell components (50, 69). These factors can activate toll-like receptor (TLR) signaling both in beta cells and macrophages by elevating circulating levels of TLR ligands, triggering signaling pathways driven by NFκB and STAT1, which consequently decrease beta cell insulin gene expression and secretion, and highlights the important role macrophage activation may play in the initiation and progression of insulitis in diabetes (34, 50, 60). There is also evidence for other hypotheses regarding the initial trigger of T1D such as a combination of genetic predisposition and environmental factors, including viruses, and it is possible that these triggers may lead to macrophage activation and the associated inflammatory cascade (61, 89).

Single-cell sequencing studies in pancreatic islets from NOD mice revealed the presence of five main subsets of macrophage populations expressing specific markers with both pro and anti-inflammatory phenotypes (90, 91). The authors suggested that two subsets called Mac-1(Apoe) and Mac-5(Stmn1) represented resident-like macrophages, possibly involved in regulating islet function (90, 91). Mac-2 macrophages (*Tnf, Ccl3, Atf3*) exhibit a pro-inflammatory profile, producing cytokines that promote T-cell activation and may contribute to islet inflammation, while Mac-3 cells (expressing *Cxcl9)* form an intermediate antigen-presenting subset that may bridge the steady-state and activated states (90). Mac-4 macrophages constitute a specialized efferocytosis subset (called e-Macs) characterized by expression of *Cd9, Lgals3 (Galectin-3), Mertk*, and *Igf1 (*91). These e-Macs emerge following limited β-cell apoptosis, perform active efferocytosis, and undergo an anti-inflammatory reprogramming that includes IGF-1 secretion (91). Functionally, e-Macs promote local immune tolerance by inducing an anergic-like phenotype in CD4^+^ T cells through the IGF-1–IGF1R axis, thereby restraining autoimmune responses and delaying diabetes onset in NOD mice (91).

Recent transcriptomic characterization of human pancreatic CD206- and CD206+ macrophages showed a gene profile quite consistent with M1 and M2 phenotype (92). CD206+ macrophages had a similar gene expression profile between exocrine and islet regions, while CD206- macrophages presented regional differences that could be related to a different ontogeny (92). Only a few differentially expressed genes were identified when comparing patients with normoglycemia, elevated HbA1c, or T2D, but the low number of patients studied, the arbitrary differentiation of macrophages based on CD206 expression, and other technical details may be influencing those results. Interestingly, the only patient studied with T1D had significantly lower levels of CD206+ macrophages (92). More studies are needed to define the different subsets of pancreatic macrophages functionally and phenotypically and to understand differences in their functions and environment interaction based on their origin. This is challenging due to their high plasticity, the existence of many intermediate states, the limited number of markers that have been identified to differentiate between functional states, and the difficulty of investigating these cells in the human pancreas, largely stemming from the limited availability of human pancreatic tissue. Thus, in humans, the mechanisms underlying the crosstalk between macrophages and beta cells, both in homeostasis and disease initiation or progression, are still largely uncharacterized.

In summary, pancreatic macrophages play a key role in disease initiation and progression in both T1D and T2D. Their functional state significantly affects insulin sensitivity, beta-cell function, immune responses, and interactions within the immune system (35). Many questions still remain, for instance, their specific location and distribution in the chronology of the disease, their intricate connections and cross-talk with other cell types in the pancreas, and their effects on the tissue microenvironment through the differential cellular networks they can establish based on their activation status. Understanding the full spectrum of activation and polarization states of macrophages in the human pancreas, including the role of newly defined subsets of macrophages, is critical to advance our knowledge in diabetes pathogenesis. The dynamic and heterogeneous pancreatic microenvironment influences macrophage polarization, often driving a pro-inflammatory state relevant to diabetes progression. A deeper understanding of the chronology of the events that locally occur in T1D and T2D is critical for designing future treatment strategies. Investigating these microenvironments and cell-to-cell interactions across different stages in disease will be crucial for identifying the role of macrophages in the development of diabetes and for designing therapeutic interventions to restore the pancreas to a healthy functional state (50, 93).

## 6 Macrophages in adipose tissue

Adipose tissue macrophages (ATMs) are the most abundant class of immune cells in the inflamed adipose tissue and are key mediators of adipocyte dysfunction and fibrosis in obesity. They play a significant role in the regulation of metabolic homeostasis, including local angiogenesis, adipose tissue remodeling, and therefore contribute to the development of complications associated with obesity (94). ATMs are highly heterogeneous, occupying distinct anatomical niches and performing distinct functions (**Table 1**). In normal human adipose tissue, macrophages account for approximately 10% of the cells. However, this proportion can soar to nearly 40% in obese individuals (30, 95). Macrophages are the primary source of TNF-α in adipose tissue, and their accumulation positively correlates with adiposity, indicating a significant contribution to the inflammatory environment (30). Additionally, in rabbits, induced overexpression of M-CSF, which is the primary regulator of macrophage differentiation and survival, is associated with a 16-fold increase in adipose tissue growth, suggesting that macrophages play a role in adipocyte hyperplasia and the physiological regulation of adipose tissue growth (31).

The activation of ATM is influenced by factors such as the presence of free fatty acids, adipokines, and other inflammatory mediators derived from adipose tissue. However, the link between adipose tissue inflammation and insulin resistance is complex. Shimobayashi et al. (96) demonstrated that insulin resistance itself can drive inflammation in adipose tissue, rather than inflammation always preceding insulin resistance. Impaired insulin signaling in adipocytes leads to increased production of pro-inflammatory cytokines and recruitment of immune cells, establishing a feed-forward loop between insulin resistance and inflammation. This highlights a bidirectional relationship between these processes (96).

Obesity can skew the adipose tissue to secrete adipokines, including MCP-1, TNF-α, IL-1β, and IL-6, which perpetuate a low-grade inflammation or meta-inflammation. MCP-1 acts through the CCR2 receptor to recruit circulating monocytes that eventually differentiate into mature macrophages (97–100). While MCP-1 is an important factor in macrophage recruitment, other mechanisms are also involved, as the absence of MCP-1 does not completely prevent monocytes from entering the adipose tissue (101). The number of macrophages can soar 40% within adipose tissue during obesity and it is positively associated with the aggravation of metabolic syndrome and the release of pro-inflammatory mediators such as IL-1β, IL-18, TNF-α, Nos2, and IL-6, which contribute to the development of insulin resistance and ultimately T2D (49, 102, 103). These proinflammatory cytokines interfere with insulin signaling, impair glucose uptake in adipocytes and recruit more monocytes and immune cells depending on environmental stimuli (104). Additionally, these recruited monocyte-derived macrophages can be retained in the adipose tissue by netrin 1, a neuroimmune guidance cue expressed in obese tissues and induced by palmitate, further promoting their accumulation (105). Proliferating macrophages often appear in crown-like structures, characterized by the presence of dead adipocytes surrounded by a ring of adipose tissue macrophages (53, 106). Collectively, these elements contribute to low-grade inflammation, a characteristic of the metabolic dysfunction that can lead to insulin resistance and the onset of T2D (56). Despite a large body of evidence suggesting that inflammation contributes to insulin resistance and T2D in mice, clinical evidence in humans has been less consistent. For example, studies have demonstrated that weight-loss induced improvements in clinical measures of insulin sensitivity have not always been accompanied by reductions in ATM content or inflammation, inflammatory genes (i.e. IL-1β, TNF-α, and CCL2) are not associated with T2D in human genome-wide association studies, and anti-inflammatory therapies have failed to improve insulin sensitivity or glycemic control in clinical trials (107, 108). This evidence highlights the need for a deeper understanding of the mechanisms driving T2D in humans, particularly the role of macrophages and inflammation in disease initiation and insulin sensitivity.

Very recently, Yu et al. (109) identified three anatomically and functionally distinct macrophage subsets in white adipose tissue; parenchymal (pATMs), capsular (cATMs), and septal (sATMs), each occupying discrete niches. sATMs, marked by *CD209b* and *LYVE1*, were long-lived, embryonically derived cells residing in the adipose septum, where they physically interact with CD26^+^ adipocyte stem cells (ASCs). Through localized *TGFβ1* signaling, sATMs instructed ASC differentiation toward white adipocytes, thereby promoting energy-storing adipogenesis and restraining thermogenic functions. Genetic depletion of sATMs or selective deletion of *Tgfβ1* in resident macrophages redirected ASC fate toward “baige” adipocytes, enhanced thermogenesis, improved insulin sensitivity, and conferred resistance to diet-induced obesity. Similar CD206^+^LYVE1^+^ human sATMs were identified in obese patients, underscoring the evolutionary conservation of this macrophage-stem cell niche (109).

Obesity is a well-known major risk factor for the development of T2D, but T1D has been traditionally considered a disease of “lean” people. However, scientific evidence indicates that obesity is strongly associated with a higher risk of developing autoimmune diseases (110). Furthermore, obesity in early childhood is associated with an increased risk of developing T1D (111). Children at risk of developing T1D with persistently elevated BMI have a 63% higher risk of developing T1D (after adjusting for age, sex, and antibody number) than children without persistently elevated BMI (112). Although direct evidence linking obesity and T1D remains limited, it is known that expanded adipose tissue can modulate ATM function as described above, leading to insulin resistance and placing greater secretory demands on beta cells (12). This added strain can promote autoimmunity through mechanisms such as beta cell stress, cytokine release, neoepitope antigen formation, and increased beta cell apoptosis, finally leading to T1D onset (113, 114). Obesity, accompanied by insulin resistance, further complicates the clinical management of T1D patients (12).

In summary, obesity promotes chronic low-grade inflammation or meta-inflammation, characterized by increased macrophage infiltration into adipose tissue, a shift in macrophage polarization toward proinflammatory states, and an overall inclination of the immune system toward inflammation (115, 116). These features are relevant for both T1D and T2D. Together, these findings highlight the complexity of ATM biology and reinforce the need for therapeutic strategies targeting pathogenic macrophage functions without compromising their protective functions.

## 7 Targeting macrophages as a therapeutic intervention in T1D and T2D

Conventional treatments for both T1D and T2D revolve around managing blood glucose levels and reducing the complications associated with poor disease control. In T1D, insulin therapy is essential because the body fails to produce enough insulin due to the high rates of beta cell destruction (117). For T2D, lifestyle modifications, oral medications to improve insulin sensitivity, and insulin may be prescribed to regulate blood sugar levels (118). However, these treatments often fail to address the underlying factors contributing to the development and progression of diabetes (119). Recently, macrophages as regulators of inflammation and insulin resistance have gained attention as potential targets for emerging therapeutics for diabetes. By targeting macrophages, the aim is to modulate their activity and reduce chronic inflammation, which is a hallmark of diabetes (120). These targeted therapeutics may hold promise not only for improving glucose control but also for addressing the underlying immune dysfunction and inflammation associated with diabetes (**Table 2**).

**Table 2:** Therapeutic strategies for targeting macrophages in T1D and T2D.

Disease	Drug/therapy	Main target(s)	Summary	Reference
T1D, T2D	Insulin	Insulin Receptor	Insulin replacement activates downstream signaling to manage blood glucose levels when the body fails to produce insulin	(117, 118)
T2D	Lifestyle modifications	AMPK, GLUT, PPAR, etc.	Regulate blood glucose through modulation of cellular pathways involved in insulin sensitivity, glucose metabolism, and overall metabolic health	(118, 141)
T1D, T2D	Macrophage Modulation	Macrophages	Targeting macrophages aims to reduce chronic inflammation, addressing immune dysfunction in diabetes	(141)
T1D, T2D	Adoptive transfer	M2 macrophages	Transfusion of M2-polarized macrophages protects against islet and renal injury in diabetic mouse models	(141, 147)
T2D	Metformin	AMPK activation, ROS inhibition	Beyond its conventional use, metformin exhibits anti-inflammatory properties, promotes M2 polarization of macrophages, and reduces hepatic gluconeogenesis, pro-inflammatory cytokines, and inflammation.	(122–125, 127, 128)
T2D	SGLT2 inhibitor (empagliflozin)	SGLT2	While generally anti-inflammatory, empagliflozin may upregulate IL-1β expression in macrophages and improve beta-cell function	(129–131)
T2D	GLP-1 based therapies	GLP-1 agonists, DPP-4 antagonists	Protective effects by decreasing macrophage infiltration and promoting M2 polarization	(133)
T2D	Thiazolidinediones (TZDs; such as rosiglitazone)	PPARγ Pathway	Induces PPARγ expression, mediating inflammation, lipid metabolism, and glutamine metabolism in macrophages	(134–137)
T1D, T2D	Anti-TNF-α Antibodies	TNF-α	May improve insulin signaling, decrease proinflammatory signaling	(155, 156)
T1D, T2D	IL-1β Inhibitors (Anakinra, Canakinumab, Xoma 052)	IL-1β	Promising results in improving glycemic control and β-cell function in clinical trials	(157–165)
T2D	IL-6 Inhibitor (Tocilizumab)	IL-6	Inhibiting IL-6 shows potential in improving insulin resistance, decreasing pro-inflammatory signaling in macrophages, and addressing T2D	(166)
T1D, T2D	recombinant IL-10 (cytokine targeting)	IL-10 activation	Associated with M2 macrophages, IL-10 improves insulitis and insulin resistance; improves pancreatic beta-cell function in response to glucose *in vitro*	(171, 172, 175, 176)
T1D, T2D	microRNA (miRNA) modulation	Various miRNAs (e.g., miRNA-155, mir-690, miR-34a, miR-146a-5p)	miRNA-155 blockage decreases M1 (pro-inflammatory) polarization; miRNA-690 mimics may alleviate glucose intolerance and insulin resistance in obese mice; could inhibit inflammation and diabetes progression	(180–187)
T1D, T2D	Other targeted therapeutics	Folate receptor	Functional folate receptor is induced during macrophage activation and can be used to target drugs to activated macrophages; folate receptor inhibition decreases insulin resistance	(188)
T1D, T2D	Other targeted therapeutics	CCR5	CCR5 inhibition may decrease obesity-induced adipose tissue inflammation and shift to M2 population via recruitment and polarization to anti-inflammatory phenotype; protects against diabetes in obese mice	(189)
T1D, T2D	Other targeted therapeutics (Iron regulation)	H-ferritin gene, iron regulation pathway	Deletion of H-ferritin may alleviate obesity and diabetes induced by high-fat diet in mice. Decreased macrophage iron levels may suppress inflammatory response and prevent diet-induced diabetes in mice	(192–195)
T1D, T2D	RNAi, Gene editing	Various Pathways (e.g., PI3K/AKT, NOTCH1, PRRs)	Targeting specific pathways using RNA interference and gene editing shows promise in decreasing inflammatory gene signaling	(196–199, 203)
T1D, T2D	RNAi, Gene editing	IRF5	Promotes inflammation resolution and improves healing through transcription regulation	(198, 200–202)
T2D	RNAi, Gene editing	IKKB inhibition (part of IKK/NFkB pathway)	Improves obesity-induced insulin resistance and reduces TNF-a production in animal models and in T2D	(204–207)
T1D, T2D	Epigenetic modifications	HDAC inhibitors, DNMT inhibitors, Jmjd3/UTX and BET Inhibitors, BAF60a	Modulating macrophage functions through epigenetic modifications may decrease inflammation and improve insulin secretion; reduces TNF production and promotes anti-inflammatory macrophages, potentially leading to islet regeneration	(209–217)
T2D	Metabolic modulation	Fatty acid synthesis	Prevents diet-induced insulin resistance, macrophage recruitment to adipose tissue, and chronic inflammation	(122, 208)
T1D	Educator therapy/direct transplantation	delivery of ex vivo macrophages, TAMEMs	“Educated” myeloid cells derived from stem cells release exosomes that facilitate the differentiation of monocytes into anti-inflammatory macrophages, offering potential control of autoimmunity and improving outcomes in T1D; prevents T1D in NOD mice	(218–221)
T1D, T2D	Nanoparticle delivery systems	Macrophage membrane disguises	Reduction of inflammation	(227)

### 7.1 Current Therapies Affecting Macrophage Function

Various currently used therapies for diabetes have effects on macrophage polarization and inflammation. Metformin is the most widely-used drug for T2D, and it has shown pleiotropic effects beyond its conventional mechanism (121) to reduce hepatic gluconeogenesis (122). It has been found to reduce pro-inflammatory cytokine levels, suppress inflammation, and promote M2 polarization of macrophages through various mechanisms, such as AMPK activation and ROS inhibition (121, 123–127). Many studies have reported the anti-inflammatory properties of metformin and the SGLT2 inhibitor, empagliflozin. Arefin et al. (128) indicated that empagliflozin may upregulate IL-1β expression in macrophages, potentially leading to increased IL-1β secretion. This observation could explain the improvement in pancreatic beta cell function and glucose sensitivity observed in the EMPA-REG BASALTM trial (129–131). GLP-1-based therapies, including GLP-1 agonists and DDP-4 inhibitors, have also demonstrated protective effects by decreasing macrophage infiltration in lesions and promoting M2 polarization (132, 133). Thiazolidinediones (TZD) compounds induce PPARγ expression and mediate inflammation, lipid metabolism, and glutamine metabolism in macrophages (134–136). TZD, such as rosiglitazone and pioglitazone, are effective insulin-sensitizing drugs that have been approved for clinical treatment of T2D. Thus, the PPARy pathway is an important target for promoting protection from insulin-resistant states and the development of diabetes (137, 138). These drugs exhibit therapeutic benefits in diabetes by modulating macrophage activity and promoting an anti-inflammatory environment, as well as by directly improving insulin sensitivity in critical tissues. However, their risk of severe adverse effects, such as congestive heart failure, myocardial infarction, and bladder cancer, among others, is a safety concern that needs to be taken into account when prescribing those drugs, especially for long-term use (139, 140).

### 7.2 Novel therapeutic strategies targeting macrophages

*i. Modulating Macrophage Polarization*.

Several strategies have been proposed for targeting macrophages for therapeutic purposes. Modulation of macrophage polarization is one such approach (141). Murine M2-like macrophages in adipose tissue play a critical role in the maintenance of insulin sensitivity and glucose homeostasis (56, 119, 135, 142, 143). Studies have shown that transfusion of M2-polarized macrophages can protect against islet and renal injury in diabetic mouse models (144). Also, genetic targeting of c-Jun-N-terminal kinases protects against insulin resistance and the switch towards an M1-like state in mice (145), as they play a role in polarization and interfere with insulin signaling (146). In humans, targeting macrophages in patients with diabetic complications, such as diabetic nephropathy and diabetic foot ulcers, shows promise in limiting monocyte recruitment, protecting against diabetes-induced injuries, and promoting wound healing through various mechanisms that promote anti-inflammatory macrophage phenotypes (147–151).

*ii. Targeting Cytokine Production*.

Targeting cytokine production and surface markers in macrophages is another approach for therapeutic intervention (141). Pro-inflammatory cytokines such as TNF-α, IL-1β, and IL-6 produced by macrophages contribute to insulin resistance and inflammation in diabetes (137, 152, 153). Inhibition of TNF-α has shown improvement in insulin sensitivity and diabetic wound healing in animal models (119, 154). However, clinical trials using anti-TNF-α antibodies have yielded mixed results (155, 156). Clinical trials using IL-1β inhibitors, such as anakinra, canakinumab, and Xoma 052, have shown promising results in improving glycemic control and beta cell function in patients with T2D (119, 137, 157, 158), ClinicalTrials.gov identifier: NCT00303394 (159), ClinicalTrials.gov identifier: NCT01327846 (160, 161). However, a systemic review of the safety and efficacy of anti-IL-1 targeted therapies, including Anakinra, Canakinumab, Gevokizumab, and Rilonacept, showed no positive treatment effects on preserving pancreatic islet function and endogenous insulin production in patients with T1D (162–164), as reviewed in (165), highlighting the need for further research to clarify their therapeutic potential in T1D or T2D (119).

IL-6 has been classically considered a pro-inflammatory cytokine involved in both innate and adaptive immune responses that is secreted by macrophages, and it has been associated with the development of T2D (119). Inhibiting IL-6 with antibodies showed potential in improving insulin resistance and T2D (119, 166). However, it is currently well appreciated that IL-6 is a pleiotropic cytokine, and it has both pro-inflammatory and anti-inflammatory effects, with complex roles in inflammation and metabolic disease. For instance, it has been recently described that IL-6 is constitutively expressed by human beta and alpha cells, suggesting a physiological role for this cytokine within the islets (167). Furthermore, expression of IL-6 was reduced in islets of donors with T1D (167) and it has been shown that IL-6 couples autophagy to antioxidant response, reducing reactive oxygen species (ROS) in beta cells, leading to stress adaptation and reducing cellular apoptosis, thereby protecting human islets from inflammatory stress-induced apoptosis (168, 169). These findings were further reinforced when a clinical trial with the IL6-receptor monoclonal antibody antagonist, tocilizumab, failed to protect beta cell loss in patients with recent onset T1D (170).

IL-10 is an anti-inflammatory cytokine associated with M2 macrophages that improves insulitis and insulin resistance in preclinical studies (171, 172). IL-10 gene transfer studies have demonstrated protective effects against autoimmune diabetes in mice (173, 174), and recombinant IL-10 has proven safe in clinical trials to treat some autoimmune diseases, with evidence that it enhances pancreatic beta cell response to glucose *in vitro* (175–177). Despite its potential, some findings complicate the therapeutic landscape for IL-10. Chronic treatments of diabetic mice with IL-10-receptor neutralizing antibodies resulted in unexpected deleterious effects on cerebral microcirculation and cognitive function in models of T1D (178). Also, IL-10 hyporesponsiveness has been implicated in chronic inflammation associated with T2D (179).

Overall, the results using anti-cytokine therapies in T1D and T2D have been controversial highlighting the complexity of the disease and the fact that cytokines can exert dual effects in inflammation and have different effects systemically and locally.

*iii. MicroRNA-Based Therapies*.

Macrophages also secrete microRNAs (miRNA) via extracellular vesicles, which contribute to insulin resistance and inflammation. Restoring aberrantly regulated miRNAs in T2D using miRNA mimics or inhibition has been proposed as a therapeutic strategy for diabetes (180–182). For example, targeting miRNA-155, which promotes M1 polarization, or mir-690, which helps alleviate glucose intolerance and insulin resistance in obese mice, could inhibit inflammation and diabetes progression (183–185). Inhibiting MiR-34a may be a future therapeutic approach, as adipocyte-to-macrophage delivery of MiR-34a inhibits Kruppel-like factor 4 (KLF4) expression, preventing M2 polarization and promoting obesity-induced insulin resistance (186). Regarding diabetes complications, exosomal miR-146a-5p from umbilical cord-derived MSCs protects against diabetic kidney disease in rats by inducing M2 macrophage polarization via targeting tumor necrosis factor receptor-associated factor 6 (TRAF6) and STAT1 (187).

*iv. Targeting Macrophage-Driven Insulin Resistance*.

It is possible that selectively impairing the function of folate receptors expressed on activated macrophages, which are involved in insulin resistance, could be a viable approach to treat diabetes (119, 188). In addition, CCR5 plays a role in the recruitment of adipose tissue macrophages and the development of insulin resistance. CCR5 loss decreases adipose tissue inflammation and shifts the macrophage population to an M2-dominant, thereby protecting against diabetes in obese mice (189). Dual CCR2/CCR5 antagonism ameliorates insulin resistance and inflammation in high-fat diet-fed mice and decreases CCL2/CCL4-induced migration of macrophages (190), however there were multiple off-target effects and so this may not be the best option for human therapy (191). Finally, deletion of the macrophage gene H-ferritin leads to decreased macrophage iron levels and a suppressed inflammatory response, preventing diet-induced diabetes in mice (192–195).

*v. RNA Interference*.

Manipulating macrophage phenotypes by targeting specific pathways using RNA interference and gene-editing techniques has also shown promise as a therapeutic strategy to treat diabetes. By targeting specific pathways, such as the PI3K/AKT and NOTCH1 pathways, as well as pattern recognition receptors, inflammatory gene signaling in macrophages can be decreased (196–199). Silencing IRF5, a transcriptional regulator in macrophages (200–202), through RNAi also has the potential to promote inflammation resolution and improve healing of diabetic wounds (196, 203). Inhibition of IKKB, a component of the IKK/NF-κB pathway, has been shown to improve obesity-induced insulin resistance and reduce TNF-α production in animal models (204, 205) and in T2D (137, 206, 207). FAS, another possible target pathway, is involved in inflammation, as it prevents diet-induced insulin resistance, macrophage recruitment to adipose tissue, and chronic inflammation (146, 208). Currently, the use of siRNA is limited by the need for safe and effective targeted delivery systems and siRNA stability.

*vi. Epigenetic Modification*.

Epigenetic modification has recently emerged as a potential strategy for modulating macrophage function in diabetes. Epigenetic modifications not only regulate inflammation but also demonstrate roles in metabolic reprogramming and ameliorating mitochondrial dysfunction (209). Histone deacetylase (HDAC) inhibitors have shown promise in decreasing the inflammatory response mediated by TLR and in improving insulin secretion in diabetic rats (210–212). Specific inhibitors of Jmjd3/UTX and BET have demonstrated the ability to reduce TNF production and promote anti-inflammatory macrophages, potentially leading to islet regeneration in T1D (213). These mechanisms of action have potential applications in T2D as well (214). Chromatin remodeling has also been studied; Kong et al. show that BAF60a, a subunit of the switch/sucrose-nonfermentable chromatin remodeling complexes, interacts with the transcription factor Atf3 to regulate ATM inflammation activation and insulin resistance in WAT, and that overexpression of BAF60a attenuates activation of pro-inflammatory macrophages in mice (215). DNA methylation via DNA methyltransferase inhibitor (DNMTi) drugs such as decitabine alleviated insulin resistance in obese mice through promoting M2 polarization and targeting PPARy (216, 217). However, further research is needed to develop effective delivery systems and ensure the safety and efficacy of these epigenetic modulators in clinical settings (146).

*vii. Adoptive Macrophage Transfer/Educator Therapy*.

Lastly, the delivery of *ex vivo* macrophages has shown promise in the treatment of diabetes, including as stem cell educator therapy and during direct transplantation (218–221). Educator therapy involves harvesting stem cells or myeloid cells such as monocytes from a patient’s blood, “educating” or reprogramming them outside the body with specific antigens or immunomodulatory signals, and reintroducing them to direct and reshape immune responses. In educator therapy, “educated” myeloid cells derived from stem cells release exosomes that facilitate the differentiation of monocytes into anti-inflammatory macrophages, offering potential control of autoimmunity and improving outcomes in T1D (218). In the context of diabetic wounds, macrophages often fail to switch from an inflammatory to an anti-inflammatory state, resulting in persistent inflammation (196, 222). Tumor-associated macrophage-educated macrophages have demonstrated reparative and immunosuppressive functions in murine diabetic wounds (220). Furthermore, the transfer of *in vitro*-induced M2 macrophages has shown promise in preventing T1D in NOD mice (219), while the transfusion of M2-polarized macrophages has also shown protective effects against islet and renal injury in diabetic mouse models (144). It is also possible that monocytes could be differentiated into anti-inflammatory M2 macrophages using educator therapy with exosomes to alleviate autoimmunity in pancreatic islets (221). Stem cells educator therapy in clinical trials of patients with T1D and T2D has had some positive results in improving metabolic control, restoration of islet beta cell function or islet beta cell regeneration, reversing autoimmunity, and improving insulin sensitivity (218, 221, 223–225). Recent data reveals that IL-1-beta levels decreased in patients treated with stem cell educator therapy, supporting its utility in T1D (221). These findings highlight the potential of *ex vivo* macrophages and alternative approaches as therapeutic strategies for diabetes and its complications. Currently, however, few educator therapies are under investigation that directly target the modulation of islet macrophages for the treatment of T1D or T2D. Recent literature reported 11 clinical trials involving macrophage cell therapy-related trials, most of which involved adoptive transfer of macrophages and *ex vivo* polarization; however, none were for diabetes treatment (226).

## 8 Conclusions

Macrophages are innate immune cells which play an important role for maintaining homeostasis and driving disease pathology. In diabetes, the main biological mechanisms underlying their pathogenic role differ in T1D and T2D. However, an increasing body of evidence suggesting that low-grade chronic inflammation participates in the initiation of T2D, and that metabolic disturbances are also relevant in T1D, indicates that there may be some common pathways as well. Macrophages play a dual role in influencing both autoimmune and metabolic responses. They are highly heterogeneous, and can dynamically switch between pro-inflammatory and anti-inflammatory states, a key feature of their ability to drive pathogenic mechanisms. In the adipose tissue, interactions between macrophages and other cellular components can have significant systemic implications. In addition, tissue resident macrophages have a crucial role in the control of organ development and tissue homeostasis.

Targeting macrophages as a therapeutic option for T1D and T2D holds promise in addressing the underlying immune dysfunction and inflammation associated with the disease. Their influence in the tissue microenvironment through multiple cellular processes, from inflammation to tissue repair and fibrosis, underscores the interest in fine-tuning macrophage activity within the disease milieu. However, challenges such as off-target effects, delivery barriers, and potential immunosuppression must be addressed before these therapeutics can be integrated into practice. In fact, despite their promise in preclinical studies, there have been limited clinical trials for macrophage-targeted therapies. Their function and their balance in tissue homeostasis are very complex, and many gaps in knowledge need to be addressed. Additionally, further research is needed to fully understand the mechanisms and long-term effects of targeting macrophages in diabetes treatment. Newly available technology may advance our understanding of the pancreatic cellular landscape and the complex interplay between cellular crosstalk involving macrophages and pancreatic function. A deeper knowledge of macrophage subsets, their plasticity, and their contribution to disease initiation and progression will allow us to design better macrophage-targeting therapies, that can be tailored to specific tissues and effectively treat human conditions. Future research should focus on delineating the precise mechanisms by which macrophages influence distinct stages of diabetes, and on unraveling the complex signaling networks that regulate macrophage behavior, paving the way for innovative therapies aimed at modulating their activity for disease prevention, intervention and improved metabolic health.
